# Synthesis and Inhibition Evaluation of New Benzyltetrahydroprotoberberine Alkaloids Designed as Acetylcholinesterase Inhibitors

**DOI:** 10.3389/fchem.2019.00629

**Published:** 2019-09-18

**Authors:** Bruna R. de Lima, Juliana M. Lima, Jéssica B. Maciel, Carolina Q. Valentim, Rita de Cássia S. Nunomura, Emerson S. Lima, Hector H. F. Koolen, Afonso Duarte L de Souza, Maria Lúcia B. Pinheiro, Quezia B. Cass, Felipe Moura A. da Silva

**Affiliations:** ^1^Central Analítica-Centro de Apoio Multidisciplinar, Universidade Federal do Amazonas, Manaus, Brazil; ^2^Separare, Departamento de Química, Universidade Federal de São Carlos, São Carlos, Brazil; ^3^Faculdade de Farmácia, Universidade Federal do Amazonas, Manaus, Brazil; ^4^Departamento de Química, Universidade Federal do Amazonas, Manaus, Brazil; ^5^Grupo de Pesquisa em Metabolômica e Espectrometria de Massas, Universidade do Estado do Amazonas, Manaus, Brazil

**Keywords:** bioaffinity chromatography, molecular docking, on-flow assay, *Onychopetalum amazonicum*, stepholidine derivatives

## Abstract

Secondary metabolites from natural products are a potential source of acetylcholinesterase inhibitors (AChEIs), which is a key enzyme in the treatment of many neurodegenerative diseases. Inspired by the reported activities of isoquinoline-derivative alkaloids herein we report the design, one step synthesis and evaluation by capillary enzyme reactor (ICER) of benzyl analogs (**1a**–**1e**) of the tetrahydroprotoberberine alkaloid stepholidine, which is abundant in *Onychopetalum amazonicum*. Docking analysis based on the crystal structure of *Torpedo californica* AChE (*Tc*AChE) indicated that π-π interactions were dominant in all planned derivatives and that the residues from esteratic, anionic and peripheral subsites of the enzyme played key interaction roles. Due to the similarities observed when compared with galantamine in the AChE complex, the results suggest that ligand-target interactions would increase, especially for the *N*-benzyl derivatives. From a series of synthesized compounds, the alkaloids (7*R*,13a*S*)-7-benzylstepholidine (**1a**), (7*S*,13a*S*)-7-benzylstepholidine (**1b**), and (*S*)-10-*O*-benzylstepholidine (**1d**) are reported here for the first time. The on flow bioaffinity chromatography inhibition assay, based on the quantification of choline, revealed the *N*-benzylated compound **1a** and its epimer **1b** to be the most active, with IC_50_ of 40.6 ± 1 and 51.9 ± 1 μM, respectively, and a non-competitive mechanism. The proposed approach, which is based on molecular docking and bioaffinity chromatography, demonstrated the usefulness of stepholidine as a template for the design of rational AChEIs and showed how the target-alkaloid derivatives interact with AChE.

## Introduction

The Amazon rainforest is considered the largest natural reservoir of plant diversity and the most diverse ecosystem on the planet (Oliveira and Amaral, 2004). Among this wide biodiversity, numerous species belonging to the Annonaceae family stand out due to their use in traditional medicine, including the treatment of neurodegenerative diseases (Adams et al., 2007), and as promising sources of bioactive natural products, such as *Onychopetalum amazonicum* (Almeida et al., 1976; Silva et al., 2015; Lima et al., 2016, 2019).

Plant natural products (PNPs) have attracted the interest of many researchers around the world due to their chemical diversity and biochemical specificity, which make them favorable as lead structures for drug discovery (Cragg et al., 1997; Harvey, 2008). Notoriously, many of these PNPs (e.g., galantamine and huperzine A) have been used in semisynthetic procedures which are designed by rational modifications in order to increase their biological activity or reduce side effects (Högenauer et al., 2001; Atanasova et al., 2015). Among these promising compounds, alkaloids are highlighted due to their high structural diversity and wide range of biological activities, including Anti-HIV (Kashiwada et al., 2005), anticancer (Lu et al., 2012), and acetylcholinesterase (AChE) inhibition (Tsai and Lee, 2010; Hostalkova et al., 2019).

In regards to AChE, this enzyme has been an attractive target for rational drug design and the discovery of mechanism-based inhibitors for the treatment of central nervous system (CNS) and peripheral diseases, such as myasthenia gravis (Cui et al., 2015), glaucoma (Almasieh et al., 2013), schizophrenia (Patel et al., 2010) and Alzheimer's disease (Murray et al., 2013). Thus, the use of AChE inhibitors (AChEIs) is considered a therapeutically-relevant strategy for these diseases (Houghton et al., 2006; Mukherjee et al., 2007; Anand and Singh, 2013; Mohammad et al., 2017), and justifies the search for new leads. Since the identification of galantamine as a powerful AChEI, the prospection of AChEIs has been boosted in the field of chemistry of natural products (Houghton et al., 2006; Mukherjee et al., 2007; Anand and Singh, 2013; Deka et al., 2017), and, several alkaloid classes, including isoquinolines, indoles, quinolizidines, piperidines, and steroidal alkaloids, have been reported as promising AChEIs (IC_50_ < 50 μM) (Murray et al., 2013). Regarding isoquinoline-derived alkaloids, recent studies pointed out berberine and tetrahydroprotoberberine salts as potent AChEIs and therefore, suitable templates to design rational AChE ligands (Tsai and Lee, 2010; Hostalkova et al., 2019).

To meet this end, complementary approaches commonly used in drug discovery are often combined, such as virtual screening and co-crystallization experiments. These allow understanding of how AChE interacts with ligands at the active site, and thus support the design and screening of new AChEIs. As an example, the determination of the crystal structure of *Torpedo californica* AChE (*Tc*AChE) permitted the visualization, at atomic resolution, of the active site of AChE that is unexpectedly located at the bottom of a deep gorge lined largely by aromatic residues (Dvir et al., 2010), suggesting that π-π stacking interactions may be of great relevance to the action of AChEIs, which corroborates the docking results for benzylated compounds and *Tc*AChE (Yamamoto et al., 1994).

Regarding the *in vitro* biological assays, a variety of colorimetric methods based on the Ellman's reagent (Ellman et al., 1961) with either free (Mantoani et al., 2016) or immobilized enzyme (Andrisano et al., 2001; Vilela et al., 2014) have been described in order to identify AChEIs in natural or synthetic libraries. The main drawback of these assays is that they are based on indirect AChE activity measurement and thus prone to false positive and/or negative results. To overcome these problems, an on-flow assay based on the use of AChE immobilized capillary enzyme reactors (AChE-ICERs) has been adopted to directly measure the production of choline (Ch). One of the key advantages of this approach is the use of a mass spectrometer as the detector, which allows differentiation between the ligands from the substrate and reaction products (Vanzolini et al., 2013; Seidl et al., 2019). The usefulness of the AChE-ICERs assay platform has been demonstrated not only as a tool for identifying inhibitors, but also for characterizing inhibition mechanisms (Vanzolini et al., 2013; Sangi et al., 2014; Torres et al., 2016; Seidl et al., 2019).

Herein, we report the design, one-step synthesis, and evaluation by ICER of new benzyl analogs obtained from stepholidine, an isoquinoline-derived neuroprotective alkaloid, which was obtained from the Amazonian plant *O. amazonicum*. The virtual screening associated to the inhibition AChE-ICER assay enabled us to pinpoint the main target-alkaloid interactions.

## Materials and Methods

### General Apparatus and Chemicals

Optical rotations were acquired at a Polartronic H-series polarimeter at the sodium D line (589 nm) and 25°C (Schmidt + Haensch, Berlin, Germany). Mass spectrometry data for structural determination were obtained using a triple quadrupole (QqQ) (TSQ Quantum Access, San Jose, CA, USA) and a quadrupole time-of-flight (Q-TOF) (Impact HD, Bruker Daltonics, Bullerica, MA, USA) mass spectrometer, both equipped with an electrospray ion source (ESI), in the positive mode. The biological assay was carried out in a chromatography system that consisted of two LC-20AD pumps, a SIL 20A autosampler, a DGU-20A5 degasser, a CTO-20A column oven and a CBM-20A interface (Shimadzu, Kyoto, Japan). The LC system was coupled to an Esquire 6000 ion trap (IT) mass spectrometer equipped with an ESI source (Bruker Daltonics, Bremen, Germany). The Data Analysis software (Bruker Daltonics, Bremen, Germany) was employed for the data acquisition. One-dimensional (1D) and two-dimensional (2D) nuclear magnetic resonance (NMR) spectroscopy data were acquired using an AVANCE III HD 500 spectrometer (Bruker, Billerica, USA) operating at 11.7 T (500.13 and 125.76 MHz for ^1^H and ^13^C, respectively). Chemical shifts (δ) were presented in ppm relative to the tetramethylsilane (TMS) signal at 0.00 ppm as an internal reference and the coupling constants (*J*) were given in Hertz. Deuterated methanol (CD_3_OD, 99.8%), was obtained from Cambridge Isotope Laboratories (Tewksbury, MA, USA). Semi-preparative high performance liquid chromatography (HPLC) analysis was performed on a Shimadzu UFLC system (LC-6 AD pump; DGU-20A5 degasser; SPD-20AV UV detector; rheodyne injector; CBM-20A communication module) (Columbia, MD, USA) equipped with a Luna C18(2) column (250 × 10 mm, 5 μm) (Phenomenex–Torrance, CA, USA). Column chromatography (CC) was carried out on silica gel 60 (230–400 mesh; Merck) and KP-C18-HS cartridge (Biotage, VA, USA). All solvents used for chromatography and MS experiments were HPLC grade and were purchased from J. T. Baker (Phillipsburg. NJ, USA), and the water was purified by using a Milli-Q system (Millipore, Bedford, MA, USA). The analytical reagents, ammonium acetate, potassium hydroxide (KOH), dimethylformamide (DMF), benzyl bromide (BnBr), AChE from *Electrophorus electricus* (*eel*AChE) type VI-S, choline iodide (Ch), acetylcholine iodide (ACh), and galanthamine bromide were purchased from Sigma-Aldrich (St. Louis, MO, USA).

### Plant Material

*Onychopetalum amazonicum* R. E. Fr. (Annonaceae) leaves were collected from a specimen previously cataloged during the Flora project (Ribeiro et al., 1999) in March, 2014 in the Adolpho Ducke Forest Reserve (26 km North on the AM-010 highway, in the municipality of Manaus, Amazonas state, Brazil, 2°59′15.9″S, 59°55′35.5″W). The access to genetic heritage was registered at Sistema Nacional de Gestão do Patrimônio Genético e do Conhecimento Tradicional Associado (SisGen) under the code #AE0F182. A voucher (#218341) was deposited in the herbarium of the Instituto Nacional de Pesquisas da Amazônia (INPA). The material was immediately dried at ambient temperature (ca. 20°C) during 20 days.

### Extraction, Synthesis, and Isolation

To obtain a stepholidine-rich fraction (SRF), the dried and powdered leaves of *O. amazonicum* (300 g) were directly subjected to an acid-base extraction (Soares et al., 2015) to give an alkaloid-rich fraction (1.36 g). Then, an aliquot of the alkaloidal fraction (1 g) was subjected to silica gel CC eluted with hexane-ethyl acetate-methanol (30:40:30, v/v), which provided 13 fractions. These fractions were pooled according to MS analysis to provide the SRF (fractions 3–8) (0.44 g), and this sample was submitted to MS, ^1^H NMR analysis, and synthesis procedures.

The compounds **1a−1e** were prepared by following an adapted one-step synthetic method (Karimova et al., 2014). The SRF (0.44 g) was added to a round bottom flask containing KOH (0.56 g) and DMF (10 mL). The resulting mixture was stirred and heated (40°C) for 30 min and then benzyl bromide (1.2 mL) was added. After 24 h, the mixture was partitioned with distilled water (20 mL) and dichloromethane (DCM) (20 mL). The DCM fraction (DCMF) (0.7 g) was dried under a nitrogen gas stream, while the aqueous fraction (AF) (1.4 g) was freeze-dried.

An aliquot (0.50 g) of the AF and DCMF was subjected to C18 CC eluted with gradient systems of water-methanol, affording 4 and 3 fractions, respectively. Fractions coded as AF3 (66.4 mg) and DCMF3 (133.1 mg) were subjected to further purification by semi-preparative HPLC using a C18 column with a constant flow-rate of 3.5 mL/min and UV detection at 235 and 280 nm. Formic acid aqueous solution (1%, v:v) (A) and methanol (B) were used as mobile phases. The gradient elution was as follows: 0–15 min, 20–50% B, 15–27 min, 50–80% B, 27–37 min, 80% B (v:v). AF3 (3 × 20 mg) and DCMF3 (6 × 20 mg) fractions were carried onto the column in water and DMSO (100 μL), respectively. The fractions coded as AF3-1 (19.6 mg–**1b**), AF3-2 (14.8 mg–**1a**), DCMF3-6 (15.3 mg–**1c**), DCMF3-7 (13.5 mg–**1d**), and DCMF3-11 (18.5 mg–**1e**) were submitted to HRMS, 1D and 2D NMR analysis.

**(7*R*,13a*S*)-7-benzylstepholidine (1a)**: yellow powder (14.8 mg); ${\left[\alpha \right]}_{D}^{25}$ = −33.88° (*c* 0.072, MeOH); ^1^H NMR (500 MHz, CD3OD) δ 3.27 (m, 1H), 3.42 (m, 1H), 3.72 (m, 1H), 3.80 (m, 1H), 3.84 (s, 3H), 3.93 (s, 3H), 4.01(dd, *J* = 17.9, 5.6 Hz, 1H), 4.29 (m, 2H), 4.33 (m, 1H), 4.60 (d, *J* = 15.7 Hz, 1H), 5.26 (dd, *J* = 12.2, 5.9 Hz, 1H), 6.78 (s, 1H), 6.96 (s, 1H), 7.03 (d, *J* = 8.3 Hz, 1H), 7.12 (d, *J* = 8.3 Hz, 1H), 7.27 (m, 2H), 7.49 (m, 2H), 7.54 (m, 1H); ^13^C NMR (125.76 MHz, CD3OD) δ 25.0, 29.9, 52.4, 56.6, 58.3, 58.5, 61.1, 68.9, 112.8, 113.5, 119.4, 121.3, 122.3, 122.8, 123.2, 126.1, 128.3, 130.7, 132.1, 133.8, 145.2, 148.0, 150.1, 150.4; HRMS *m/z* 418.2011 (calcd. for C_26_H_28_NO_4_, 418.2018, Δ_m/z*theoretical*_ = −1.67 ppm).

**(7*S*,13a*S*)-7-benzylstepholidine (1b)**: yellow powder (19.6 mg); ${\left[\alpha \right]}_{D}^{25}$ = −35.10° (*c* 0.082, MeOH); ^1^H NMR (500 MHz, CD3OD) δ 3.15 (dd, *J* = 18.1, 10 Hz, 1H), 3.33 (m, 1H), 3.42 (dd, *J* = 18.1, 10 Hz, 1H), 3.50 (m, 1H), 3.57 (m, 1H), 3.81 (s, 3H), 3.85 (m, 1H), 3.91 (s, 3H), 4.52 (d, *J* = 15.7 Hz, 1H), 4.66 (m, 2H), 4.71 (d, *J* = 15.7 Hz, 1H), 4.74 (m, 1H), 6.78 (s, 1H), 6.80 (d, *J* = 8.3 Hz, 1H), 6.85 (d, *J* = 8.3 Hz, 1H), 6.96 (s, 1H), 7.50 (m, 2H), 7.54 (m, 2H), 7.57 (m, 1H); ^13^C NMR (125.76 MHz, CD3OD) δ 24.2, 35.3, 51.7, 56.7, 56.9, 60.9, 65.0, 65.5, 113.5, 114.4, 118.9, 120.7, 120.8, 121.8, 125.0, 125.8, 128.4, 130.7, 132.3, 134.4, 145.7, 147.7, 150.2, 150.3; HRMS *m/z* 418.2001 (calcd. for C_26_H_28_NO_4_, 418.2018, Δ_m/z*theoretical*_ = −4.06 ppm).

**(*S*)-2-*O*-benzylstepholidine (1c)**: yellow powder (15.3 mg); ${\left[\alpha \right]}_{D}^{25}$ = −387.60° (*c* 0.26, MeOH); ^1^H NMR (500 MHz, CD3OD) δ 2.71 (dd, *J* = 16.1, 11.4 Hz, 1H), 2.80 (m, 1H), 2.87 (m, 1H), 3.12 (ddd, *J* = 16.1, 11.2, 5.4 Hz, 1H), 3.37 (m, 2H), 3.74 (d, *J* = 15.6 Hz, 1H), 3.82 (s, 3H), 3.83 (s, 3H), 4.40 (d, *J* = 15.6 Hz, 1H), 5.09 (s, 2H), 6.75 (s, 1H), 6.76 (d, 8.3 Hz, 1H), 6.80 (d, 8.3 Hz, 1H), 6.90 (s, 1H), 7.29 (m, 1H), 7.37 (m, 2H), 7.44 (m, 2H); ^13^C NMR (125.76 MHz, CD3OD) δ 28.6, 35.7, 52.4, 54.3, 56.5, 60.4, 72.6, 113.3, 113.5, 116.9, 125.4, 126.2, 127.1, 127.6, 128.9, 129.1, 129.4, 138.7, 145.0, 148.2, 149.1, 150.4; HRMS *m/z* 418.2010 (calcd. for C_26_H_28_NO_4_, 418.2018, Δ_m/z*theoretical*_ = −1.91 ppm).

**(*S*)-10-*O*-benzylstepholidine (1d)**: yellow powder (13.5 mg); ${\left[\alpha \right]}_{D}^{25}$ = −146.02° (*c* 0.42, MeOH); ^1^H NMR (500 MHz, CD3OD) δ 2.74 (m, 1H), 2.79 (m, 2H), 3.10 (m, 1H), 3.34 (m, 1H), 3.38 (dd, *J* = 16.3, 4.1 Hz, 1H), 3.65 (m, 2H), 3.82 (s, 3H), 4.3 (d, *J* = 15.6 Hz, 1H), 5.08 (s, 2H), 6.68 (s, 1H), 6.75 (s, 1H), 6.90 (d, *J* = 8.5 Hz, 1H), 6.97 (d, *J* = 8.5 Hz, 1H), 7.30 (m, 1H), 7.37 (m, 2H), 7.45 (m, 2H); ^13^C NMR (125.76 MHz, CD3OD) δ 29.0, 36.2, 52.9, 54.7, 56.5, 60.8, 60.9, 72.1, 112.7, 113.3, 115.1, 125.3, 125.9, 128.0, 128.6, 128.8, 129.1, 129.7, 129.8, 138.8, 146.5, 147.0, 148.3, 151.0; HRMS *m/z* 418.2009 (calcd. for C_26_H_28_NO_4_, 418.2018, Δ_m/z*theoretical*_ = −2.15 ppm).

**(*S*)-*O***, ***O*-dibenzylstepholidine (1e)**: yellow powder (18.5 mg); ${\left[\alpha \right]}_{D}^{25}$ = −96.15° (*c* 0.32, MeOH); ^1^H NMR (500 MHz, CD3OD) δ 2.71 (m, 1H), 2.65 (m, 2H), 3.08 (m, 1H), 3.21 (m, 1H), 3.25 (m, 1H), 3.52 (m, 2H), 3.82 (s, 3H), 3.86 (s, 3H), 4.21 (d, *J* = 16.1 Hz, 1H), 5.02 (s, 2H), 5.08 (s, 2H), 6.71 (s, 1H), 6.85 (d, *J* = 8.2 Hz, 1H), 6.86 (s, 1H), 6.92 (d, *J* = 8.2 Hz, 1H), 7.30 (m, 2H), 7.36 (m, 4H), 7.44 (m, 4H); ^13^C NMR (125.76 MHz, CD3OD) δ 29.1, 36.3, 52.5, 54.7, 56.5, 60.6, 60.7, 71.9, 113.3, 113.6, 114.7, 125.1, 128.3, 128.6, 128.8, 128.91, 129.0, 130.2, 138.8, 146.8, 148.0, 150.1, 150.7; HRMS *m/z* 508.2473 (calcd. for C_33_H_34_NO_4_, 508.2488, Δ_m/z*theoretical*_ = −2.95 ppm).

### Molecular Docking

The docking studies were carried out according to a previously reported approach (Santos et al., 2016). First, the 3D structures of stepholidine and compounds **1a−1e** were generated and checked in relation to the protonated state in pH 7.4, and the tautomers via Marvin Sketch (ChemAxon, 2017). Then, to identify the optimized structures with the lowest energy, the structures were treated by the semi-empirical method PM7 (Stewart, 2013) using MOPAC2016 software (Stewart, 2016). The refined structures were converted into PDBQT files via Autodock tools (Morris et al., 2009). The three-dimensional crystal structure of *Tc*AChE complexed with galantamine was retrieved from the RCSB (Research Collaboratory for Structural Bioinformatics) protein data bank (http://www.rcsb.org) under PDB ID 1QTI (Bartolucci et al., 2001). The receptor preparation was as previously reported (Santos et al., 2016), in which the grid box was centered at the ligand to cover the entire binding site. Finally, a rigid docking process was carried out using Autodock Vina (Trott and Olson, 2010) with Discovery Studio (AccelrysInc, 2016) being used to analyze the binding conformations.

### Biological Evaluation Based on AChE-ICER Assay

AChE from *Electrophorus electricus* (type VI-S) was immobilized in a fused-silica capillary tube (*eel*AChE-ICER) according to a previously reported protocol (Vanzolini et al., 2013). An enzyme solution containing 2 units/mL *eel*AChE was used in the immobilization procedure. The LC-MS system was set up for the *eel*AChE-ICER as previously described (Vanzolini et al., 2013). The mobile phase (15 mM ammonium acetate solution, pH 8.0) was infused by pump 1 at a flow rate of 50 μL/min and pump 2 delivered acetonitrile after the *eel*AChE-ICER at a flow rate of 50 μL/min. IT-MS parameters were: 3,713 V capillary voltage, 500 V end plate voltage, 7.0 mL/min drying gas, 300°C drying temperature and 30 psi nebulizer. In addition, ACh ([M+H]^+^ *m/z* 146) and Ch ([M+H]^+^ *m/z* 104), were analyzed by MS operating in the manual MS^*n*^ mode under positive ionization (scan 50–550 *m/z*). Enzymatic activity was quantified using a Ch standard external calibration curve prepared in 15 mM ammonium acetate solution, pH 8.0 with the final concentration ranging from 5 to 350 μM. An aliquot (10 μL) of each solution was injected in triplicate into the system using an empty fused silica capillary. The calibration curve was obtained by plotting the Ch [M+H]^+^ *m/z* 104 area against the injected Ch concentration.

The derivatives compounds **1a−1e**, stepholidine and galantamine were screened with *eel*AChE-ICER. Stock solutions of each compound were prepared in methanol at 1.0 mM. The enzymatic activity of *eel*AChE-ICER was measured in the presence and absence of each compound. Reactions mixtures were prepared by mixing 20 μL of ACh stock solution (350 μM, in 15mM ammonium acetate solution, pH 5.0) with 10 μL of methanol or stock solution of compounds (1.0 mM) and 70 μL ammonium acetate solution (15mM, pH 8.0). Each reaction mixture (10 μL) was injected into the LC-MS system. The percentage of inhibition (%I) was calculated by comparing the product peak area in the presence of tested compound with positive control (absence of inhibitors).

Inhibitory potency (IC_50_) for galantamine and compounds (**1a** and **1b**) was obtained by directly quantifying Ch production in the presence of different concentrations of inhibitors. Stock solutions of galantamine (50–4,000 μM), derivatives **1a** and **1b** (both at 10–5,000 μM) were prepared in methanol. Reactions mixtures were prepared by mixing 20 μL of ACh stock solution (500 μM, in 15 mM ammonium acetate solution, pH 5.0) with 10 μL of stock solution of galantamine, **1a** or **1b**. Final volumes were completed with 70 μL ammonium acetate solution (15mM, pH 8.0) and each reaction mixture (10 μL) was injected into the LC-MS system. The inhibition curve was obtained for each sample by plotting the percentage of inhibition vs. each corresponding inhibitor concentration, and the IC_50_ values were achieved by non-linear regression analysis using GraphPad Prism 5 software.

The mechanism of action and its steady-state inhibition constant (K_i_) were also determined for derivatives **1a** and **1b**. For this purpose, the *eel*AChE-ICER activity was evaluated under different concentrations of ACh (10 to 150 μM) and at the fixed concentration of derivative **1a** and **1b** (0, 10, 50, and 75 μM). Lineweaver-Burk plots were used to determine the action mechanism for the inhibitors.

K_i_ values for **1a** and **1b** were determined from the slope of the Lineweaver-Burk plots vs. the respective derivative concentration. A linear replot was obtained, and the division quotient between the linear and angular coefficients provided the Ki values.

## Results and Discussion

### Molecular Docking of *N*-benzyl and *O*-benzyl Stepholidine Derivatives

The ligand binding pocket of *Tc*AChE is heavily hydrophobic and largely lined by aromatic residues, thus suggesting that an increase in *in vitro* potency could be, theoretically, achievable by the accommodation of compounds with favored π-π stacking interactions in the binding pocket. Therefore, a preliminary docking study was conducted with the tetrahydroprotoberberine alkaloid stepholidine, a neuroprotective natural product, abundant in the Amazonian species *O. amazonicum* (Yang et al., 2007; Hao et al., 2015; Lima et al., 2019) and a series of its *N*-benzyl and *O*-benzyl derivatives. This was achieved by comparison of their binding energy and main interactions in the crystallographic structure with the galanthamine-*Tc*AChE complex, since this model has already been successfully applied in virtual screening approaches of AChEIs (Rollinger et al., 2004, 2009).

**Figure 1:**
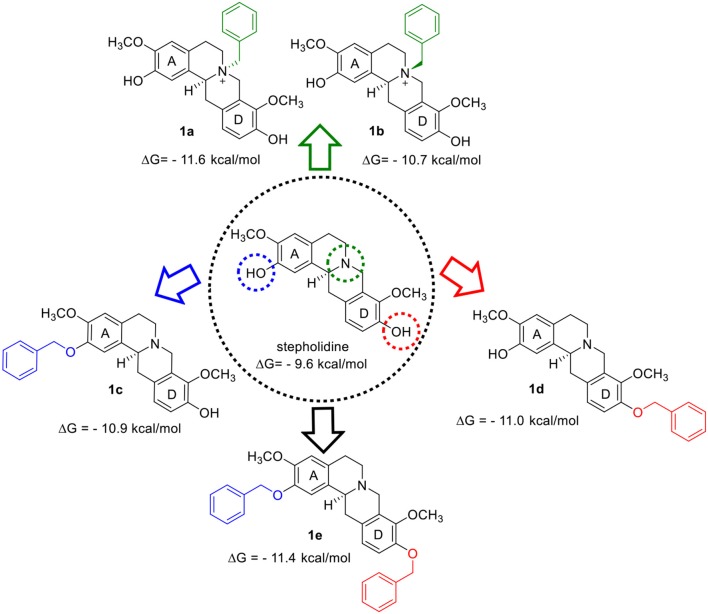
Design of the AChEIs from the benzylation of stepholidine.

Compounds **1a−1e** (Figure 1) presented better scoring function values than stepholidine and galantamine (redocking binding free energy = −9.6 kcal/mol, RMSD < 2), which suggests the establishment of new favorable interactions for the ligands-*Tc*AChE complex. Since the active-site gorge of *Tc*AChE contains two subsites, esteratic and anionic, which correspond to the catalytic machinery (Ser200, Glu327 and His440) and the choline-binding pocket (Trp84 and Phe330), respectively, we therefore focused our interpretation on these interactions, in addition to the interactions in peripheral subsite (Trp279) (Dvir et al., 2010).

**Figure 2:**
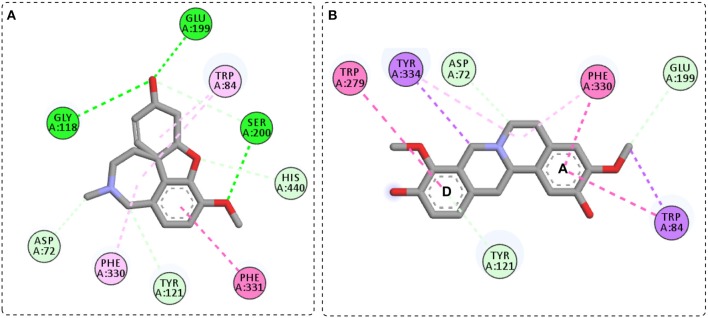
General interactions for galantamine-*Tc*AChE **(A)** and stepholidine-*Tc*AChE **(B)** complex.

Here, the observed interactions for galantamine (Figure 2A) were similar to the one described by Bartolucci et al. (2001). The oxygen atom of the *O*-methyl group participated in hydrogen bonding with Ser200 from the esteratic subsite, while its hydroxyl oxygen formed hydrogen bonds with residues Gly118 and Glu199. Also, π-alkyl interactions were observed with Trp84 and Phe330 at the anionic subsite. On the other hand, stepholidine showed π-π stacking interactions of A and D aromatic rings, respectively with the anionic (Trp84 and Phe330) and peripheral subsites (Trp279) (Figure 2B).

**Figure 3:**
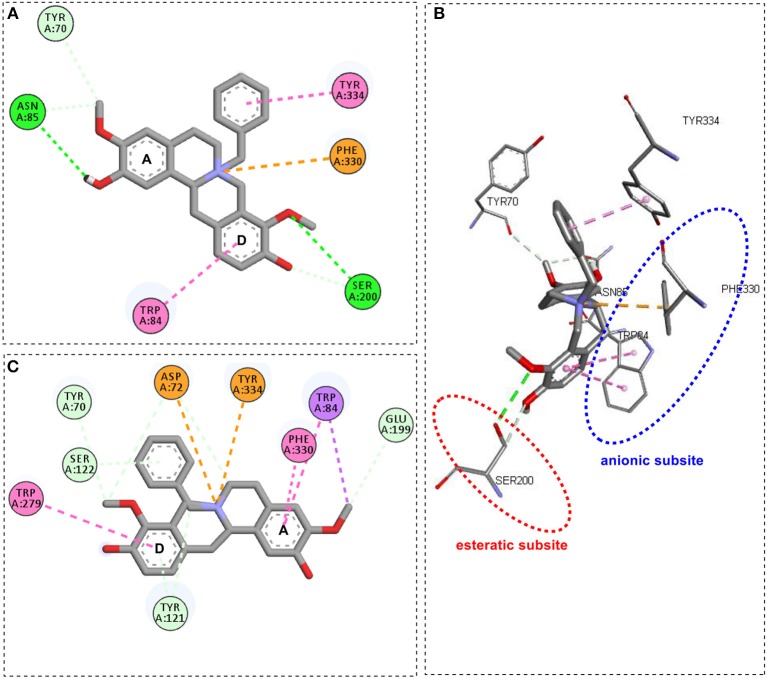
General interactions for compound **1a**-*Tc*AChE complex by 2D diagram **(A)** and 3D view **(B)**, and compound **1b**-*Tc*AChE complex by 2D diagram **(C)**.

Compound **1a**, a *N*-benzyl derivative, showed that the oxygen atom of the *O*-methyl group from the D ring established a hydrogen bonding with Ser200 from esteratic subsite (Figures 3A,B), similar to galantamine. Besides, the aromatic D ring and the positively charged nitrogen presented π-π stacked and π-cation interactions with Trp84 and Phe330 from the anionic subsite, respectively, which contrasts with the π-alkyl interactions from galantamine. In addition, π-π interaction was observed between the benzyl moiety and the Tyr334, and hydrogen bond interactions between the hydroxyl oxygen from the A aromatic ring with Asn85. On the other hand, compound **1b**, an epimer of **1a**, presented π-π stacked interactions between the A and D aromatic ring and the anionic (Trp84 and Phe330) and peripheral subsites (Trp279) (Figure 3C), similarly to stepholidine. Besides this, the positively charged nitrogen presented π-cation and attractive charge interactions with Asp72 and Tyr334, respectively. Surprisingly, no relevant interaction was observed for the benzyl moiety.

**Figure 4:**
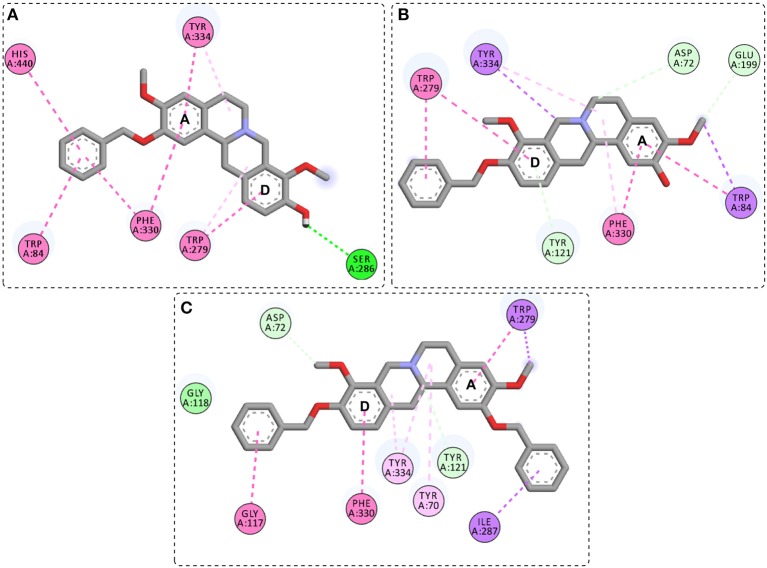
General interactions between compound **1c (A)**, **1d (B)**, and **1e (C)**, and the *Tc*AChE enzyme by 2D diagram.

Compound **1c**, an *O*-benzyl derivative, presented several π-π type interactions (Figure 4A), highlighting those between benzyl moiety from A ring and Trp84, Phe330 and His440, therefore they comprise part of the catalytic machinery and the choline-binding pocket of *Tc*AChE. The A ring also showed π-π interactions with the residues Phe330 and Tyr334. In addition to the Trp279 interaction in the D ring, also observed in the stepholidine and compound **1b**, a hydrogen bond between the hydroxyl oxygen and Ser286 was observed. Compound **1d**, another *O*-benzyl derivative, displayed similar interactions with stepholidine, except for the π-π stacked between the benzyl moiety from D ring and Trp279 from the peripheral subsite (Figure 4B). On the other hand, for compound **1e**, an *O, O*-dibenzyl derivative, the interactions of Phe330 and Trp279 with the A and D aromatic rings (Figure 4C) were reversed in relation to compounds **1b**, **1d** and stepholidine. Also, for the benzyl moieties, π-sigma and π-π stacked interactions were observed with Ile287 and Gly117, respectively.

Taking into account the docking analysis, it can be perceived that π-π interactions are dominant in compound series **1a−1e** with key interactions with residues from esteratic, anionic and peripheral subsites. It was also construed that the *N*-benzyl derivatives, given their interaction similarities with galantamine in the *Tc*AChE complex, would favor the activity.

**Scheme 1:**
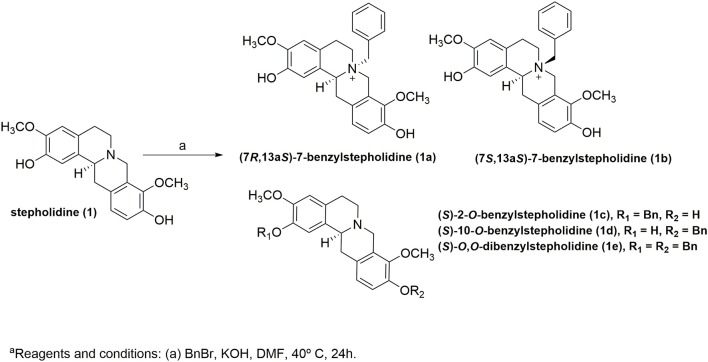
One-step synthesis of compounds **1a−1e**^a^.

Remarkably, the stepholidine *N*-benzyl and *O*-benzyl derivatives showed higher scoring values than either galantamine or stepholidine. Therefore, compounds **1a−1e** were synthesized following reaction steps described at Scheme 1, followed by full characterization and, then, submitted to inhibition screening.

### Structural Determination

The MS and ^1^H NMR spectra of SRF (Supplementary Material) displayed signals that were consistent with the stepholidine structure, which is in agreement with a recently published study about the *O. amazonicum* alkaloid content (Lima et al., 2019). On the other hand, the stepholidine concentration in the SRF was determined as being >85% based on the integration of the aromatic signals in the ^1^H NMR spectrum. To evaluate the stereochemistry of the 13a position in the stepholidine and of compounds **1a−1e**, stepholidine previously isolated from *O. amazonicum* (Lima, 2005) was subjected to polarimetric analysis, and presented ${\left[\alpha \right]}_{D}^{25}$ = −231.69° (*c* 0.19, MeOH), this value was in accordance with the 13a*S* configuration (Chen and Yang, 2009).

Compound **1a** was obtained as a yellow amorphous powder. Its molecular formula was determined as C_26_H_28_NO_4_ by HRMS (obs. *m/z* 418.2011; calcd. 418.2018, Δ_m/z*theoretical*_ = −1.67 ppm). The MS/MS spectrum of the ion at *m/z* 418 presented two main neutral losses of 92 (*m/z* 326) and 150 Da (*m/z* 268), which are consistent with a tetrahydroprotoberberine alkaloid containing methoxyl and hydroxyl groups at D ring (Lima et al., 2019) and an *N*-benzyl group (Kuck et al., 2011). Ratifying the MS analysis, the ^1^H NMR exhibited signals of an *ortho*-substituted ring at δ_H_ 7.12 (d, *J* = 8.3 Hz, 1H) and 7.03 (d, *J* = 8.3 Hz, 1H), as well signals of a *para*-substituted ring at δ_H_ 6.96 (s) and 6.78 (s), and two methoxyl signals at δ_H_ 3.93 (s) and 3.84 (s), all typical signals of the stepholidine structure (Lima et al., 2019), while the benzyl signals were observed at δ_H_ 7.54 (m, 1H), 7.49 (m, 2H), 7.27 (m, 2H), and 4.29 (d, *J* = 4.9 Hz, 2H). The confirmation of the *N*-benzylation was achieved via the heteronuclear multiple bond correlation (HMBC) experiment. In this, *J*^3^-couplings for the benzyl methylene protons at δ_H_ 4.29 (m, 2H) with the carbons at δ_C_ 52.4 and 58.3 confirmed our hypothesis. The stereochemistry of the 13a position was assigned as *S* based on the precursor stereochemistry, and a negative value was also observed in the polarimetric analysis (${\left[\alpha \right]}_{D}^{25}$ = −33.88°, *c* 0.072, MeOH). Due to the observation of a nuclear Overhauser effect (NOE) between the benzyl methylene protons at δ_H_ 4.29 and the methine proton at δ_H_ 5.26 (dd, *J* = 12.2, 5.9 Hz, 1H) in the nuclear Overhauser enhancement spectroscopy (NOESY) spectrum, the stereochemistry of the nitrogen atom was confirmed as *R*. Therefore, compound **1a** was determined as being the previously undescribed tetrahydroprotoberberine alkaloid named (7*R*,13a*S*)-7-benzylstepholidine.

Compound **1b** was obtained as a yellow amorphous powder. Its molecular formula was determined as C_26_H_28_NO_4_ by HRMS (obs. *m/z* 418.2001; calcd. 418.2018, Δ_m/z*theoretical*_ = −4.06 ppm). A comparative analysis of the MS and NMR data for compounds **1a** and **1b** indicated the same structure. Since they were separated via HPLC, it was assumed that **1b** was a diastereomer of **1a**. Similarly, for compound **1a**, the stereochemistry of the 13a position for compound **1b** was assigned as *S* based on the precursor stereochemistry, and a negative value was also observed in the polarimetric analysis (${\left[\alpha \right]}_{D}^{25}$ = −35.10°, *c* 0.082, MeOH). Due to the observation of a NOE effect between the benzyl methylene proton at δ_H_ 4.66 and the methylene proton at δ_H_ 3.50 (m, 1H) in the NOESY spectrum, the stereochemistry of the nitrogen atom was confirmed as *S*. Therefore, compound **1b** was determined as being the previously undescribed tetrahydroprotoberberine alkaloid named (7*S*,13a*S*)-7-benzylstepholidine.

Compound **1c** was obtained as a yellow amorphous powder. Its molecular formula was determined as C_26_H_28_NO_4_ by HR-MS (obs. *m/z* 418.2010; calcd. 418.2018, Δ_m/z*theoretical*_ = −1.91 ppm). The MS/MS spectrum of the ion at *m/z* 418 presented two main neutral losses of 91 (*m/z* 327) and 150 Da (*m/z* 268), which are consistent with a tetrahydroprotoberberine alkaloid contain methoxyl and hydroxyl groups at D ring (Lima et al., 2019) and an *O*-benzyl group (Kuck et al., 2011). Ratifying the MS analysis, the ^1^H NMR also exhibited typical signals of the stepholidine structure. The aromatic benzyl signals were observed at δ_H_ 7.44 (m, 2H), 7.37 (m, 1H), 7.29 (m, 2H), while the methylene signals were observed at δ_H_ 5.09 (s, 2H). The confirmation of the *O*-benzylation at A ring was reached via the *J*^3^-couplings for the benzyl methylene protons at δ_H_ 5.09 (s, 2H) with the carbon at δ_C_ 148.1 in the HMBC experiment. The stereochemistry of the 13a position was assigned as *S* based on the precursor stereochemistry, a negative value was also observed in the polarimetric analysis (${\left[\alpha \right]}_{D}^{25}$ = −387.60°, *c* 0.26, MeOH). Therefore, compound **1c** was determined as the previously described tetrahydroprotoberberine alkaloid named (*S*)-2-*O*-benzylstepholidine.

Compound **1d** was obtained as a yellow amorphous powder. Its molecular formula was determined as C_26_H_28_NO_4_ by HRMS (obs. *m/z* 418.2009; calcd. 418.2018, Δ_m/z*theoretical*_ = −2.15 ppm). A comparison between the MS and NMR data of compounds **1c** and **1d** indicated that these compounds shared similar skeleton, as the position of the *O-*benzyl group was the main difference between them. The confirmation of the *O*-benzylation at D ring was achieved via the *J*^3^-couplings for the benzyl methylene protons at δ_H_ 5.08 (s, 2H) with the carbon at δ_C_ 150.9 in the HMBC experiment. The stereochemistry of the 13a position was also assigned as *S* based on the precursor stereochemistry, a negative value was also observed in the polarimetric analysis (${\left[\alpha \right]}_{D}^{25}$ = −146.02°, *c* 0.42, MeOH). Therefore, compound **1d** was determined as being the previously undescribed tetrahydroprotoberberine alkaloid named (*S*)-10-*O*-benzylstepholidine.

Compound **1e** was obtained as a yellow amorphous powder. Its molecular formula was determined as C_33_H_34_NO_4_ by HRMS (obs. *m/z* 508.2473; calcd. 508.2488, Δ_m/z*theoretical*_ = −2.95 ppm). A comparison between the MS and NMR data of compounds **1c**, **1d**, and **1e** indicated that **1e** shared similar *O*-benzyl positions with **1c** and **1d**. This was confirmed via the *J*^3^-couplings for the benzyl methylene protons at δ_H_ 5.08 (s, 2H) and 5.02 (s, 2H) with the carbons at δ_C_ 147.9 and 150.6, respectively, in the HMBC experiment. The stereochemistry of the 13a position was also assigned as being *S* based on the precursor stereochemistry, a negative value was also observed in the polarimetric analysis (${\left[\alpha \right]}_{D}^{25}$ = −96.15°, *c* 0.32, MeOH). Therefore, compound **1e** was determined as the previously described tetrahydroprotoberberine alkaloid named (*S*)-*O,O*-dibenzylstepholidine.

### Biological Evaluation Based on AChE-ICER Assay

It is well-accepted that docking studies are carried out using *Tc*AChE due to the availability of its crystal structure and the established knowledge of ligand interactions for this enzyme (Houghton et al., 2006; Mohamed and Rao, 2010; Gupta et al., 2011). In the case of inhibition assays, the use of *eel*AChE is also well-accepted as a substitute for human AChE (Mohamed and Rao, 2010; Gupta et al., 2011; Vanzolini et al., 2013). Moreover, both enzymes have conserved primary sequences in the active residues (Gupta et al., 2011). Thus, stepholidine and its derivatives **1a**–**1e** were screened by the inhibition *eel*AChE-ICER on flow assay. ACh was used as substrate, while galantamine was used as a positive control. The results of inhibition percentages are given in Table 1.

**Table 1:** Inhibition percentages of *N*-benzyl and *O*-benzyl stepholidine derivatives against *eel*AChE.

**Compound**	**% Inhibition *eel*AChE-ICER**
Galantamine	95.8 ± 1.87
Stepholidine	40.2 ± 1.65
**1a**	90.1 ± 2.04
**1b**	90.5 ± 2.48
**1c**	35.2 ± 5.02
**1d**	46.3 ± 5.45
**1e**	43.6 ± 2.54

**Figure 5:**
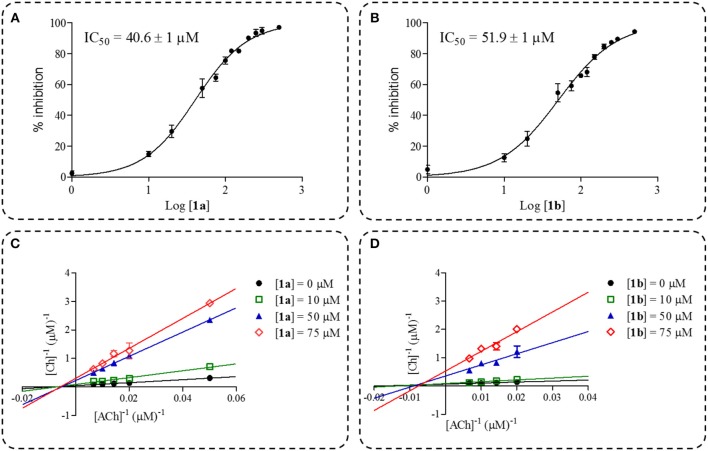
Dose-response curve plots of inhibition percentage for compound **1a (A)** and **1b (B)**. Double-reciprocal plots for compound **1a (C)** and **1b (D)**.

The screening assay disclosed compound **1a** and its epimer **1b** as the inhibitors (Table 1), with inhibition values close to those obtained for galantamine, while compounds **1c**-**1e** and stepholidine presented values below 50%. Thus, IC_50_ values were determined only for these two compounds, in which the values obtained were of 40.6 ± 1 for **1a** and 51.9 ± 1 μM for **1b** (Figures 5A,B). Regarding the increase in activity observed for compounds **1a** and **1b**, this is in accordance with a previous published study (Loizzo et al., 2008) which was carried out with stepholidine (IC_50_ > 100 μM), *N*-methyl stepholidine (IC_50_ = 31.30 μM) and stepharanine (IC_50_ = 14.10 μM), and which showed that stepholidine has significantly less activity than its quaternary derivatives. This suggests that a positive charge at the nitrogen portion can influence AChE inhibitory activity.

In spite of the IC_50_ values of compounds **1a** and **1b** being higher when compared to galantamine under the same experimental conditions (IC_50_ = 17.1 ± 1.1 μM), the mechanism of inhibition and K_i_ were determined and, both inhibitors **1a** (K_i_ = 11.6 μM) and **1b** (K_i_ = 4.7 μM) substantially reduced the rate of the enzymatic reaction, which illustrates a characteristic behavior of non-competitive inhibitors as observed in the double-reciprocal plots (Figures 5C,D).

The evaluation of inhibition modality is a relevant assessment in the early stages of drug discovery programs, since the mode of interaction could be affected, however this depends on the physiological contexts to which the enzyme is exposed. As an example, competitive inhibitors bind exclusively to the free enzyme form, while non-competitive or mixed type inhibitors bind with some affinity to both forms e.g., the free enzyme and the enzyme-substrate complex. Thus, the non-competitive inhibition modality can be a significant advantage *in vivo* when the physiological context exposes the enzyme to high substrate concentrations. Although the clinical advantage of non-competitive inhibition has been recognized, there are a very large number of drugs in clinical use today that are competitive enzyme inhibitors, and which can be related to historical approaches for drug discovery that have been focused on active site-directed inhibitors (Copeland, 2016). Here, we presented two benzyl analogs from stepholidine that act via the non-competitive mechanism. The mechanistic differences between compounds **1a**, **1b** and galantamine could be assigned to the π-π interactions with Trp84 and Phe330, which were observed only for these two stepholidine derivatives with higher inhibitory potency. These results highlight the importance of the π-π interaction for the ligands and thus can be used for designing non-competitive AChEIs, which explore the clinical advantages of this inhibition modality.

## Conclusion

The proposed approach, which is based on the design of new tetrahydroprotoberberine alkaloids with increased π-π stacking interactions in the ligand binding pocket followed by inhibition analysis through AChE-ICER assay, proved to be a useful strategy for the identification of new AChEIs. Docking analysis results suggest an increase in the inhibition potency for the *N*-benzyl and *O*-benzyl derivatives when compared to the precursor stepholidine, which was corroborated by the biological inhibition data. In addition, the biological results showed that *N*-benzyl stepholidine derivatives are more active than *O*-benzyl derivatives, which suggests that the quaternary nitrogen plays a key role in AChE inhibition. These observations, along with key interactions observed in docking analysis, can be useful in the design of new AChEIs. Overall, the proposed approach demonstrated the usefulness of stepholidine as a suitable template for the design of rational AChEIs and demonstrated how the target-alkaloid derivatives interact with AChE.

## Data Availability

All datasets generated for this study are included in the manuscript/Supplementary Files.

## Author Contributions

BL and FS were responsible for the molecular docking. BL and JM prepared the stepholidine-rich fraction. BL, CV, and EL were responsible for the synthesis procedure. BL, RN, and AS were responsible for the compounds isolation. BL, HK, FS, and MP were responsible for the characterization of compounds. BL, JL, and QC were responsible for the biological assays. BL, JL, HK, FS, and QC drafted the manuscript and all the other authors contributed to the intellectual content and revision of the manuscript.

### Conflict of Interest Statement

The authors declare that the research was conducted in the absence of any commercial or financial relationships that could be construed as a potential conflict of interest.
