# Supplementary material for: Synthesis and Inhibition Evaluation of New Benzyltetrahydroprotoberberine Alkaloids Designed as Acetylcholinesterase Inhibitors
Source: Front Chem. 2019 Sep 18;7:629. doi: 10.3389/fchem.2019.00629 (PMC6760013; doi:10.3389/fchem.2019.00629)
Supplement: Supplementary file 1 [file Table_1.DOCX]

Synthesis and Inhibition Evaluation of New Benzyltetrahydroprotoberberine Alkaloids Designed as Acetylcholinesterase Inhibitors

Bruna R. de Lima^1^, Juliana M. Lima^2^, Jéssica B. Maciel^1^, Carolina Q. Valentim^3^, Rita de Cássia S. Nunomura^1,4^, Emerson S. Lima^3^, Hector H. F. Koolen^5^, Afonso Duarte L de Souza^1,4^, Maria Lúcia B. Pinheiro^1,4^, Quezia B. Cass^2*^ and Felipe Moura A. da Silva^1*^

^1^Central Analítica - Centro de Apoio Multidisciplinar (CAM), Universidade Federal do Amazonas, Manaus, AM, Brazil

^2^ Separare, Departamento de Química, Universidade Federal de São Carlos, São Carlos, SP, Brazil

^3^Faculdade de Farmácia, Universidade Federal do Amazonas, Manaus, AM, Brazil

^4^Departamento de Química, Universidade Federal do Amazonas, Manaus, AM, Brazil

^5^Grupo de Pesquisa em Metabolômica e Espectrometria de Massas, Universidade do Estado do Amazonas, Manaus, AM, Brazil

*** Correspondence:**Dr. Felipe Moura
felipemas@ufam.edu.br

Dra. Quezia Cass

[qcass@ufscar.br](mailto:qcass@ufscar.br)

Supplementary Material

**Supplementary Figure 1.** ^1^H NMR spectrum (500.13 MHz, CD_3_OD) of stepholidine-rich fraction.

**Supplementary Figure 2.** MS spectrum of stepholidine-rich fraction.

**Supplementary Figure 3.** HRMS spectrum of compound **1a** ((7*R*,13a*S*)-7-benzylstepholidine).

**Supplementary Figure 4.** MS/MS spectrum of compound **1a** ((7*R*,13a*S*)-7-benzylstepholidine).

**Supplementary Figure 5.** ^1^H NMR spectrum (500.13 MHz, CD_3_OD) of compound **1a** ((7*R*,13a*S*)-7-benzylstepholidine).

**Supplementary Figure 6.** ^13^C NMR spectrum (125.76 MHz, CDCl3) of compound **1a** ((7*R*,13a*S*)-7-benzylstepholidine).

**Supplementary Figure 7.** HSQC spectrum (500.13 MHz, CDCl3) of compound **1a** ((7*R*,13a*S*)-7-benzylstepholidine).

**Supplementary Figure 8.** HMBC spectrum (500.13 MHz, CDCl3) of compound **1a** ((7*R*,13a*S*)-7-benzylstepholidine).

**Supplementary Figure 9.** NOESY spectrum (500.13 MHz, CDCl3) of compound **1a** ((7*R*,13a*S*)-7-benzylstepholidine).

**Supplementary Figure 10.** HRMS spectrum of compound **1b** ((7*S*,13a*S*)-7-benzylstepholidine).

**Supplementary Figure 11.** MS/MS spectrum of compound **1b** ((7*S*,13a*S*)-7-benzylstepholidine).

**Supplementary Figure 12.** ^1^H NMR spectrum (500.13 MHz, CD_3_OD) of compound **1b** ((7*S*,13a*S*)-7-benzylstepholidine).

**Supplementary Figure 13.** ^13^C NMR spectrum (125.76 MHz, CDCl3) of compound **1b** ((7*S*,13a*S*)-7-benzylstepholidine).

**Supplementary Figure 14.** HSQC spectrum (500.13 MHz, CDCl3) of compound **1b** ((7*S*,13a*S*)-7-benzylstepholidine).

**Supplementary Figure 15.** HMBC spectrum (500.13 MHz, CDCl3) of compound **1b** ((7*S*,13a*S*)-7-benzylstepholidine).

**Supplementary Figure 16.** NOESY spectrum (500.13 MHz, CDCl3) of compound **1b** ((7*S*,13a*S*)-7-benzylstepholidine).

**Supplementary Figure 17.** HRMS spectrum of compound **1c** ((*S*)-2-*O*-benzylstepholidine).

**Supplementary Figure 18.** MS/MS spectrum of compound **1c** ((*S*)-2-*O*-benzylstepholidine).

**Supplementary Figure 19.** ^1^H NMR spectrum (500.13 MHz, CD_3_OD) of compound **1c** ((*S*)-2-*O*-benzylstepholidine).

**Supplementary Figure 20.** ^13^C NMR spectrum (125.76 MHz, CDCl3) of compound **1c** ((*S*)-2-*O*-benzylstepholidine).

**Supplementary Figure 21.** HSQC spectrum (500.13 MHz, CDCl3) of compound **1c** ((*S*)-2-*O*-benzylstepholidine).

**Supplementary Figure 22.** HMBC spectrum (500.13 MHz, CDCl3) of compound **1c** ((*S*)-2-*O*-benzylstepholidine).

**Supplementary Figure 23.** HRMS spectrum of compound **1d** ((*S*)-10-*O*-benzylstepholidine).

**Supplementary Figure 24.** MS/MS spectrum of compound **1d** ((*S*)-10-*O*-benzylstepholidine).

**Supplementary Figure 25.** ^1^H NMR spectrum (500.13 MHz, CD_3_OD) of compound **1d** ((*S*)-10-*O*-benzylstepholidine).

**Supplementary Figure 26.** ^13^C NMR spectrum (125.76 MHz, CDCl3) of compound **1d** ((*S*)-10-*O*-benzylstepholidine).

**Supplementary Figure 27.** HSQC spectrum (500.13 MHz, CDCl3) of compound **1d** ((*S*)-10-*O*-benzylstepholidine).

**Supplementary Figure 28.** HMBC spectrum (500.13 MHz, CDCl3) of compound **1d** ((*S*)-10-*O*-benzylstepholidine).

**Supplementary Figure 29.** HRMS spectrum of compound **1e** ((*S*)-*O*,*O*-dibenzylstepholidine).

**Supplementary Figure 30.** MS/MS spectrum of compound **1e** ((*S*)-*O*,*O*-dibenzylstepholidine).

**Supplementary Figure 31.** ^1^H NMR spectrum (500.13 MHz, CD_3_OD) of compound **1e** ((*S*)-*O*,*O*-dibenzylstepholidine).

**Supplementary Figure 32.** ^13^C NMR spectrum (125.76 MHz, CDCl3) of compound **1e** ((*S*)-*O*,*O*-dibenzylstepholidine).

**Supplementary Figure 33.** HSQC spectrum (500.13 MHz, CDCl3) of compound **1e** ((*S*)-*O*,*O*-dibenzylstepholidine).

**Supplementary Figure 34.** HMBC spectrum (500.13 MHz, CDCl3) of compound **1e** ((*S*)-*O*,*O*-dibenzylstepholidine).
